# Germination Development of Powdery Mildew on Natural and Artificial Wheat Leaf Surfaces: A Study to Investigate Plant Wax Signals

**DOI:** 10.1002/smsc.202200092

**Published:** 2023-01-31

**Authors:** Miriam Anna Huth, Axel Huth, Lukas Schreiber, Kerstin Koch

**Affiliations:** ^1^ Faculty of Life Sciences Rhine-Waal University of Applied Sciences Marie-Curie-Str. 1 47533 Kleve Germany; ^2^ IZMB Department of Ecophysiology University of Bonn Kirschallee 1 53115 Bonn Germany

**Keywords:** artificial leaf surface, *Blumeria graminis*, chemical signaling, plant wax, *Triticum aestivum*, wettability

## Abstract

Wax chemistry, especially long‐chain aldehydes, and surface wettability are discussed to have a stimulating effect on the development of *Blumeria graminis*, the pathogen of powdery mildew on wheat. Here, these specific surface properties are investigated on leaves of different wheat varieties and on developed test systems coated with plant wax and wax components. So far, the wettability of leaves has not been achieved by artificial substrates used in in vitro studies. With the test systems developed here, a wettability comparable to that on leaf surfaces and the signal character of wax chemistry could be investigated individually and in combination. The results show that wax morphology and chemistry (analyzed by scanning electron microscopy and gas chromatography) as well as wettability of leaves have no differences that would suggest a relationship with variety susceptibility. Furthermore, the influence of wax chemistry and wettability on the process of prepenetration of *B. graminis* on leaves and test systems is investigated. Germination and differentiation are not stimulated on any of the test systems compared to surfaces without the signals offered. Wettability, wax chemistry, and components such as long‐chain aldehydes could not be identified as decisive signals for the germination development of *B. graminis*.

## Graphical abstract

Wax morphology, wax chemistry, and leaf wettability are analyzed on three different wheat varieties. In addition, various test systems are developed that mimic the properties of the plant surface individually and in combination. To investigate the influence of surface properties on fungal development, wheat leaves and the test systems are inoculated with powdery mildew conidia and germination is studied.

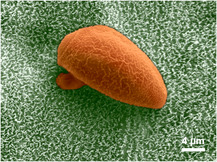

## 1 Introduction

### 1.1 Powdery Mildew

Powdery mildew fungi (Erysiphales), from the phylum Ascomycetes, can infect many different crops and wild plants.^[^ ^1^ ^]^ The disease takes its name from the white pustules, reminiscent of flour dust, that form on the surfaces of infected plants. Although the appearance is quite similar, powdery mildews are often very host specific.^[^ ^2^ ^]^ In total, there are about 650 powdery mildew species.^[^ ^3^ ^]^ One of the most economically relevant species is *Blumeria graminis*, the pathogen causing powdery mildew on grasses (Poaceae), to which all cereals belong.^[^ ^1^ ^]^ The pathogen is therefore one of the most important pathogens worldwide.^[^ ^4^ ^]^ The pathogen causing powdery mildew on wheat is *B. gramini*s f. sp. *tritici*. *B. graminis* is a fungus that can infect all aboveground plant parts. It does not kill the host plant, but growth is inhibited and quality and yield are reduced.^[^ 5, 6 ^]^ In severe cases, yield loss can be up to 40%.

Germination and differentiation of spores on the leaf surface are the first steps in pathogenesis. Conidia (asexual spores) are continuously formed by conidiophores (conidia carriers). When they are mature, they are spread predominantly by wind. After a conidium has landed on a host plant, the infection process begins. The development takes place in clearly distinguishable phases. Within seconds of contact with the plant surface, an extracellular matrix (ECM) is released by the conidia.^[^ 7, 8 ^]^ It enables conidial attachment and plays a role in substrate recognition.^[^ 9, 10 ^]^ After about 0.5–2 h, a short primary germ tube (5–10 μm) develops that adheres to the surface and forms a penetration cone to penetrate the cuticle.^[^ 11, 12, 13 ^]^ In addition to adhesion and anchoring, it serves to recognize the host surface.^[^ 14, 15 ^]^ It also allows water^[^ ^16^ ^]^ and smaller molecules to be taken up from the host cell.^[^ ^17^ ^]^ Unlike most powdery mildew species, *B. graminis* develops a second germ tube after ≈3 h, which is further differentiated.^[^ ^11^ ^]^ After this has elongated (30–40 μm), it thickens at the apical end, it is then called appressorial germ tube. After ≈9–10 h, an appressorium develops from the appressorial germ tube, which can be recognized by its hook‐shaped structure and septa formation.^[^ ^12^ ^]^ A penetration cone forms from the appressorium. Due to the activity of various enzymes, such as cutinases, and due to a high turgor pressure of up to 2–4 MPa, this can penetrate the cuticle and the cell wall.^[^ ^18^ ^]^ This completes the so‐called prepenetration process. Starting from the penetration cone, a finger‐shaped haustorium forms in the epidermal cells, through which the fungus obtains its nutrients from the plant.^[^ ^19^ ^]^ Within 24 h, the haustorium is fully developed, and the fungus forms an epiphytic mycelium on the plant surface. After three days, macroscopic white fungal colonies become visible, which in turn begin to produce conidia.^[^ ^20^ ^]^

### 1.2 The Plant Cuticle

The cuticle is the first point of contact between the fungal spores and the plant surface. The plant cuticle is a thin extracellular membrane attached to the epidermis. It forms the outermost layer of all aboveground, primary plant organs (leaves, shoots, and fruits). The plant cuticle basically consists of cutin, an insoluble polymeric matrix, and soluble hydrophobic waxes. Cutin is a biopolyester consisting mainly of unsaturated ω‐hydroxyl and epoxyl, C_16_ and C_18_ fatty acids, which gives the membrane its stability.^[^ ^21^ ^]^ Polysaccharide fibers and sometimes pectin connect the cuticle with the underlying cell wall. Among many other vital functions, protection of the plant from desiccation is the main function of the cuticle.^[^ 22, 23 ^]^ The cuticular waxes essentially provide this barrier function.^[^ 24, 25, 26 ^]^ Cuticular waxes consist of various long‐chain hydrocarbon compounds of different substance classes. The complex substance mixtures typically consist of fatty acids, alcohols, ketones, aldehydes, and triterpenes.^[^ 27, 28, 29 ^]^ The exact wax composition, however, is species‐ and organ specific.^[^ ^30^ ^]^ Plant waxes are embedded in the cutin matrix (intracuticular waxes) and are deposited on the cuticle (epicuticular waxes). In addition to their localization, intra‐ and epicuticular waxes also differ in their chemical composition.^[^ 31, 32 ^]^

Epicuticular waxes form a thin wax film (<1 μm) and often diverse 3D microstructures (0.5–100 μm) on the wax film.^[^ 33, 34, 35 ^]^ A summary of the different wax morphologies is given by Barthlott, et al.^[^ ^36^ ^]^ The most abundant structures include platelets and tubules.^[^ ^36^ ^]^ The morphology of wax structures is largely influenced by their chemical components, e.g., most platelets have a high proportion of primary alcohols.^[^ ^37^ ^]^ In 1871, De Bary^[^ ^38^ ^]^ already suggested the name wax crystals for the wax structures. The crystallinity of the epicuticular waxes was later proven by means of X‐ray structure analysis.^[^ 39, 40 ^]^ The crystallinity of the wax film, which was previously described as amorphous, was also proven.^[^ ^41^ ^]^ By recrystallizing extracted plant waxes, it could be demonstrated that the various microstructures of the epicuticular waxes develop through self‐assembly.^[^ 34, 37, 42, 43, 44, 45, 46 ^]^ In addition to wax mixtures, individual wax components were also recrystallized.^[^ 44, 47 ^]^ Thus, it could be shown that often one component is structurally decisive. For example, pure 1‐octacosanol, the main component of wheat wax, also crystallized as platelets, like the total wax.^[^ ^44^ ^]^ Through the recrystallization of plant waxes, the nanostructures of plant surfaces and the associated properties could be transferred to technical surfaces.^[^ 45, 47, 48, 49, 50 ^]^ It was thus possible to create an artificial surface that has the chemical information of the cuticular waxes and a wettability comparable to that of a leaf.^[^ ^51^ ^]^

### 1.3 Wetting

In general, the cuticle is a hydrophobic surface due to its chemical composition. However, depending on different chemical and structural variations, plant surfaces can have very different wetting properties ranging from extremely “water‐loving” (superhydrophilic) to extremely “water‐avoiding” (superhydrophobic).^[^ ^52^ ^]^ Several studies give a good review of the influence of different microstructures on the wettability of plant surfaces.^[^ 53, 54, 55, 56 ^]^ To describe the wettability of a surface, the contact angle *θ* (CA, the angle between the liquid–solid and the liquid–vapor interface) is used. The more wettable a surface is, the smaller the contact angle. In the event of complete wetting, the contact angle is 0°. In the event of wetting, a distinction is made between superhydrophilic (0°≤ *θ* < 10°), hydrophilic (10° ≤ *θ* < 90°), hydrophobic (90° ≤ *θ* < 150°), and nearly unwettable, superhydrophobic surfaces (150° ≤ *θ*).^[^ ^56^ ^]^ In addition, the contact angle hysteresis (CAH) and the tilting angle (TA) of droplets characterize the wettability of heterogeneous and rough surfaces. The CAH indicates how mobile a droplet is on a tilted surface.^[^ ^57^ ^]^ The larger it is, the harder a drop rolls off.^[^ ^58^ ^]^ It is defined as the difference between the advancing and the receding contact angle that can be measured for a drop on an inclined plane. If the hysteresis is low, drops can roll off easily without much resistance.^[^ 59, 60 ^]^ The TA α denotes the angle of inclination at which a drop begins to roll off.

### 1.4 Signals for Pathogenic Fungi

The waxes attached to the cuticle play a central role in the interaction of plants with other living organisms. The plant cuticle is therefore of particular ecological relevance.^[^ 61, 62, 63, 64 ^]^ It represents an important defence mechanism of the plant against pests such as pathogenic fungi. In addition to the mechanical barrier, the ability to self‐clean contributes to the protection of the plant.^[^ 65, 66 ^]^ In contrast, paradoxically, the plant surface also provides important physical, topographical, and chemical key stimuli that are necessary for the development of fungal pathogens.^[^ 67, 68 ^]^ The ability to recognize suitable host surfaces on the basis of such signals and to initiate the prepenetration process is one reason for the success of pathogenic fungi.^[^ ^69^ ^]^

Due to its biotrophic lifestyle, it is not possible to cultivate *B. graminis* without its host plant. However, many studies have shown that conidia can germinate on artificial surfaces. In addition to epidermis strips and isolated cuticles, the prepenetration process of the fungus could be investigated on numerous artificial surfaces in the past. For example, plastic discs, cellulose membranes, FEP film, or glass plates (coated with wax) served as substrates for germination experiments.^[^ 7, 9, 14, 15, 70, 71, 72, 73, 74, 75, 76, 77, 78 ^]^ *B. graminis* f.sp. *hordei* was mostly used as a model organism. Comparative studies with natural and artificial surfaces were thus able to show the decisive importance of the substrate on the germination success. If the primary germ tube does not perceive an inductive surface, the development of the fungus stops. The extension of the second germ tube and the formation of appressoria do not take place.^[^ ^12^ ^]^ Occasionally, several short germ tubes develop. These subsidiary germ tubes, however, are not functional.^[^ ^15^ ^]^

In many studies, artificial surfaces with different wettabilities were used to investigate the influence of wettability on germination behavior. For example, it could be shown that the conidia developed further on hydrophobic surfaces than on hydrophilic surfaces.^[^ 7, 9, 76, 79 ^]^ The germ tube on hydrophilic surfaces could not come into contact with the surface. Furthermore, no appressoria were formed.^[^ 7, 14, 70, 74 ^]^ On a hydrophobic artificial surface such as silanized glass, the formation of the appressoria was initiated.^[^ ^15^ ^]^

The chemical composition of the surface is also a decisive factor for successful germination.^[^ 80, 81 ^]^ Research is focussing on the cuticular waxes in particular. Their importance for the germination process is assessed differently. Some authors assume that the wax structures do not play a role, as the removal of the epicuticular waxes does not influence the prepenetration process.^[^ 76, 82 ^]^ In contrast, various studies could prove the inducing effect of the waxes on the prepenetration process of *B. graminis*.^[^ 75, 76, 77, 78, 83, 84, 85 ^]^ As early as 1972, Yang and Elingboe were able to demonstrate the positive effect of the waxes with the help of *cer* wax mutants and artificial surfaces, which was also emphasized by Rubiales, et al.^[^ ^86^ ^]^ after renewed germination trials with *cer* wax mutants. In German ryegrass (*Lolium perenne*), germination success depended not only on hydrophobicity but also on the chemical composition and structure of the epicuticular waxes.^[^ 78, 87 ^]^ Furthermore, studies with epidermal strips of host and nonhost plants could prove that only the wax of the host plant led to successful germination.^[^ ^75^ ^]^ Long‐chain aldehydes (C_26_, C_28_, C_30_) in particular have been shown to be signaling substances in in vitro experiments.^[^ 75, 78, 83, 84, 88, 89, 90 ^]^ In vivo, the germination‐inducing effect of the long‐chain aldehydes on the development of *B. graminis* could also be demonstrated with a wax‐free maize mutant (*glossy* 11) and with a barley mutant (*low wax* 1).^[^ 84, 85 ^]^

Zabka et al.^[^ ^76^ ^]^ and Ringelmann et al.^[^ ^78^ ^]^ showed that the wax chemistry and hydrophobicity of the surface together stimulate the prepenetration of the fungus. However, the interdependence of wax structure and chemistry with the surface wetting have only been partially considered in the past. In addition to experiments on the plant, the knowledge gained about the signaling effect of the waxes comes from experiments with test systems that only partially corresponded to the biological model. The in vitro systems used so far did not have any 3D wax structures, or they deviated from those of the leaves, so that there were inevitably wettabilities that deviated significantly from the plant surface. The differentiation rates of the fungus could be stimulated by the chemical substances, but they remained behind those on the plant surface. In addition to the wax chemistry, the wax structure and wettability were also altered in the introduced in vivo experiments. Ultimately, it remains unclear whether the wax structures, which determine wetting in addition to wax chemistry, are not also decisive for the complete germination development of the fungus.

Based on previous findings on *B. graminis* f. sp. *hordei*, this study investigates the role of the chemistry of epicuticular waxes and their wettability on the development of *B. graminis* f. sp. *tritici* conidia on artificial leaf surfaces and natural leaves. In particular, the question of whether the same wettability as on the natural surface stimulates the prepenetration process of *B. graminis* f. sp. *tritici* will be investigated. First, it was examined whether wheat varieties with different susceptibility to *B. graminis* f. sp. *tritici* show different surface properties. For this purpose, the wax composition, the wax morphology, and the wettability of the wheat leaves were analyzed. In addition, an artificial leaf surface was produced by recrystallization of wheat wax to transfer the properties of the natural surface to technical surfaces. This test system mimics both the chemical properties of the epicuticular waxes and the wettability of a leaf surface. Such in vitro test systems offer the advantage over in vivo experiments that the properties to be investigated can be modified. By changing the recrystallization parameters, further in vitro test systems were created to investigate the influence of wax chemistry and wettability individually and in combination. Finally, both the natural surfaces and the artificial surfaces were inoculated in order to investigate the germ development and to be able to identify germ‐inducing signals of the plant surface waxes. The experiments should show whether the prepenetration process of *B. graminis* f. sp. *tritici* is stimulated by the wax chemistry, by long‐chain aldehydes, by hydrophobicity, and/or by the chemical and physical factors together.

## 2 Experimental Section

### 2.1 Plant Material

Three different varieties of wheat (*Triticum aestivum*) with varying degrees of susceptibility to powdery mildew (*B. graminis* f. sp. *tritici*) were used for the experiments: The variety Ponticus (Strube D & S GmbH, Söllingen, Germany) with a very low to low susceptibility, the variety Porthus (Strube D & S GmbH, Söllingen, Germany) with a medium susceptibility, and the variety Akteur (Deutsche Saatveredelung AG, Lippstadt, Germany) with a strong to very strong susceptibility.^[^ ^91^ ^]^

The wheat plants were cultivated in the greenhouse of Rhine‐Waal University (greenhouse plants, GH). Plants were also grown outdoors in the experimental garden (outdoor plants, OD). The GH plants were grown in pots with an arable soil sand mixture, as well as universal soil (1:1:2, UV‐transparent glass, temp. day: 18 °C, temp. night: 12 °C). They were watered manually and fertilized with multiple fertilizer (2.5% Universol Blue 18‐11‐18 + 2.5 MgO + TE, Everris International BV, Heerlen, The Netherlands). The OD plants were sown by hand with a row spacing of ≈35 cm and a grain spacing of ≈10 cm. Fertilizer was applied once with saltpetre granules (YaraLiva CALCINIT, Yara GmbH & Co. KG, Dülmen, Germany). Leaves of positions 2, 3, and 4 of the plants in the 5 leaf stage were used for the experiments. The oldest leaves were in the second position and the youngest in the fourth position.

### 2.2 Development of In Vitro Test Systems

Five test systems with different physicochemical properties were developed: one test system with the same wetting properties and the same chemical properties as the wheat leaf (wax test system), one test system with the same wetting properties (C28OH test system), one test system with the same chemical properties (melt test system), and two test systems with the same wetting properties plus the potential signaling agent 1‐hexacosanal or 1‐octacosanal (C26CHO and C28CHO test systems) (**Table** **1**). The different test systems were used to investigate the influence of the imitated properties on the germination development.

**Table 1:** Developed test systems

Test systema)	Imitated property/ies of the leaf	Recrystallization of
Wax	Wetting and chemistry	Wax
C28OH	Wetting	Alcohol
Melt	Chemistry	Wax without 3D structure
C26CHO	Wetting and pot. signaling agent	Alcohol + 1‐hexacosanal
C28CHO	Wetting and pot. signaling agent	Alcohol + 1‐octacosanal

a) C28OH, 1‐octacosanol; C26CHO, 1‐hexacosanal; C28CHO, 1‐octacosanal.

#### 2.2.1 Substances Used

For the preparation of the wax test system, the wax of the wheat variety Ponticus was extracted. For this purpose, freshly cut leaves were immersed in chloroform (Rotisolv HPLC, Carl Roth, Karlsruhe, Germany) for 20 s. Finally, the extracts were filtered (ROTILABO type 113 A, 110 mm diameter, Carl Roth GmbH und Co.KG, Karlsruhe, Germany), and the resulting filtrate was dried under a fume hood until the chloroform had fully evaporated. For the C28OH test system, the pure substance 1‐octacosanol (≥99% (GC), Sigma Aldrich Co, St. Louis, USA) was used. For the melt test system, the wax of the Ponticus variety was recrystallized from the melt (see following section). For the preparation of the C26CHO and the C28CHO test system 1‐octacosanol, 1‐hexacosanal and 1‐octacosanal were used. The aldehydes were synthesized for this purpose according to Corey and Suggs (1975) from hexacosanol (≥97% (GC), Sigma Aldrich Co, St. Louis, MO, USA) and 1‐octacosanol (≥99%, GC grade, Sigma Aldrich Co, St. Louis, MO, USA) with pyridinium chlorochromate (PCC, 98%, Sigma Aldrich Co, St. Louis, MO, USA).

#### 2.2.2 Substrates Used

Glass (cover slip, 18 × 18 mm) was used as substrate for the vapor depositions. The glass was cleaned with paper moistened with ethanol (70%) and wiped dry before each vapor deposition. Epoxy resin replicas from a glass slide (following section) were used as substrate for the melt test systems, as preliminary tests had shown that no wax film could form on glass.

#### 2.2.3 Making the Replicas

Replicas are used to copy the structure of a surface without transferring its chemical properties. To mould the structures, first a negative is made, which is filled with epoxy resin to obtain a positive of the moulded structure again. The epoxy resin (Toolcraft, Conrad Electronics SE, Hirschau, Germany) used to make the replicas is a mixture of the resin and a hardener. After the resin and the hardener were added and mixed in a 10:4 ratio, the mixture was centrifuged at 600 rpm for 1 min to remove air bubbles that had formed when the substances were mixed. To produce a replica of a glass surface, a negative mould was first made from a microscope slide using a moulding material made of polyvinylsiloxane (President light body, Coltene, Altstätten, Switzerland). After the negative had hardened (≈5 min), 2–3 drops of the epoxy resin were applied to the negative with a syringe. After the resin had hardened (≈24 h at room temperature (RT)), the replicas were coated by vapor deposition (see Section 2.2.4).

#### 2.2.4 Coating of the Substrates by using Physical Vapor Deposition

The physical vapor deposition (PVD) process was used to coat the samples with the wax and individual wax components. Compared to other methods, such as recrystallization from a solution, this method has the advantage that a homogeneous surface coating can be produced. The vapor deposition was carried out with a device as described in Huth, et al.^[^ ^51^ ^]^ This previous study also demonstrated the chemical stability of the wax during the evaporation process. The device consists of a holder for the samples to be coated above a flat heater made of high‐performance ceramics (40 Ω, BACH Resistor Ceramics GmbH, Werneuchen, Germany) in a glass chamber (Vakuum‐ und Industrieservice Meier GmbH, Borken, Germany) connected to a vacuum pump (Duo 5 M, Pfeiffer Vacuum GmbH, Asslar, Germany) and a laboratory power supply unit. To evaporate the substances, they were placed on the heating plate. Then a vacuum was created, and they were heated until they evaporated. The distance between the heating plate and the samples to be coated was 4 cm.

For the wax test systems, 1400 μg of wax extract was evaporated. Since the wheat wax consists of ≈70% 1‐octacosanol, 70% of the used masses of the wax test system were used for the evaporations with 1‐octacosanol in order to obtain the same amount of 1‐octacosanol on both test systems. For the C28OH test systems, 1000 μg of the pure substance was therefore evaporated. After coating, the samples were stored at 50 °C for 3 days.

For the production of the melt test system without 3D recrystallized wax structures, the evaporated wax was melted and cooled down to interrupt the crystallization process. After evaporating 1400 μg of wax extract, the samples were heated to 100 °C overnight in an oven and then cooled down to 4 °C for this purpose.

To develop the two aldehyde test systems, 1520 μg of 1‐hexacosanal, 1160 μg of 1‐octacosanal, and 30.07 mg of 1‐octacosanol were first dissolved in 10 mL of chloroform. Subsequently, the suspensions were mixed in such a way that a comparable ratio of the substances as in the wax extract of the leaves was obtained (1‐hexacosanal: 0.5%; 1‐octacosanal: 4.2%; 1‐octacosanol: 74.7% of the total wax, see results section). The solvent of the alcohol‐aldehyde mixtures was completely evaporated under a fume hood. For recrystallization, 1000 μg of each alcohol–aldehyde mixture was used. The aldehyde test systems recrystallized for three days at 50 °C.

### 2.3 SEM Analysis of Wax Morphology

An earlier study had shown the same wax structures on leaves 2, 3, and 4 as well as on the leaves of a greenhouse and an outdoor wheat culture.^[^ ^51^ ^]^ Therefore, in this study, only the wax morphology of leaf 3 of the greenhouse culture was analyzed to compare the wax structures of the different varieties. The wax structures of the abaxial and adaxial leaf sides of the different wheat varieties and the structures on the different test systems were examined using by scanning electron microscopy (SEM, Gemini Supra 40 VP, Zeiss, Oberkochen, Germany, *n = *3).

In preparation for the SEM investigations, the middle parts of fresh wheat leaves were cut into ≈0.3 × 0.5 cm pieces with a scalpel. The wheat leaf samples and the test systems were attached to aluminum SEM stubs (diameter 2.4 cm, Plano, Wetzlar, Germany) with conductive double‐sided tape (Leit‐Tabs, Plano, Wetzlar, Germany). Subsequently, the samples were coated (sputtered) with an ≈8 nm thin gold layer for 60 s at 30 mA and 0.1 mbar using a sputter coater (Cressington 108 auto SE, Elektronen‐Optik‐Service GmbH, Dortmund, Germany) in order to increase the conductivity of the samples and to reduce charging by the electron beam during the SEM measurement. The samples were investigated at a voltage of 10 keV with the secondary electron detector with a working distance of 4–7 mm. The aperture was 30 μm.

### 2.4 Analysis of Wettability

The contact angles (CAs), CAH, and TAs of water droplets on the wheat leaves and of the developed test systems were determined using a goniometer (OCA 35, DataPhysics Instruments GmbH, Filderstadt, Germany) (*n* = 15). The CAH was measured on an inclined plane shortly before the roll‐off of the drop (tilting plate method). The leaves were tilted so that the droplets could roll longitudinally to the leaf venation. The CAs were determined in the first image of the videos of the CAH and the TA measurements. All measurements were carried out at RT with demineralized water (drop volume: 5 μL). If a droplet was rolled off the surface immediately after application, the TA was set to 0.1°. The wettability of the artificial surfaces was compared with those of wheat leaves (leaf 3) of the Ponticus variety.

### 2.5 Analysis of Wax Composition

The wax chemistry of the different varieties was compared to investigate whether there are variety‐specific differences in wax composition. In particular, the total amounts of wax, the main component (potentially structuring), and the C_26_ and C_28_ aldehyde (potentially signaling) were analyzed. To extract the waxes of the OD plants, freshly cutted leaf 2, 3, and 4 were combined together for one replicate and dipped for 20 s in 20 mL chloroform. Six independent biological replicates were analyzed (*n* = 6). After wax extraction, the leaf areas were determined immediately by scanning the leaves (LA2400 with Winrhizo, Regent Instruments Inc., Québec, Canada). To each wax extract, 50 μL of tetracosane (C_24_ alkane, *c* = 0.2 mg mL^−1^, Merck KGaA, Darmstadt, Germany) was added as an internal standard. For chemical analysis, an aliquot of each wax extract was taken, whose volumes were reduced to 200 μL at 70 °C, under a stream of gaseous nitrogen. The quantitative wax analysis was carried out with by gas chromatography coupled with a flame ionization detector (GC‐FID, 6890 N, Agilent Technologies Sales & Services, column: DB‐1; 30 m × 0.32 mm, 0.1 μm; J & W, Agilent Technologies Sales & Services GmbH & Co. KG, Waldbronn, Germany). The wax components were analyzed qualitatively by gas chromatography coupled with mass spectrometry (GC–MS, 5973, Agilent Technologies Sales & Services GmbH & Co. KG, Waldbronn, Germany). Wax monomers were fragmented by electron impact ionization and thereby identified using HP‐Chemstation software (Hewlett Packard Cooperation, Palo Alto, USA). The carrier gases were hydrogen for GC‐FID and helium for GC–MS. The sample injection volume was 1 μL.

### 2.6 Analysis of Conidia Development

To test whether there are variety‐related differences in the development of the fungus, the germination success was determined on the leaves of the three different wheat varieties with different susceptibilities to powdery mildew. Germination success was determined on the adaxial side of leaf 3 of the plants at the 5‐leaf stage. The different test systems with different chemical and physical properties were used to test the influence of the imitated parameters on germination success.

#### 2.6.1 Inoculation of the Surfaces

Powdery mildew was cultivated on wheat plants for the experiments (20 °C, 12 h/12 h day/night rhythm, 76% rel. air humidity). For establishing the culture, infested plants were provided by Prof. Ulrich Schaffrath (RWTH Aachen, Germany). Plants with powdery mildew pustules used for inoculation were always shaken manually one day before the experiment. This ensured that old conidia were removed and only newly formed conidia were transferred. To inoculate the leaves and the test systems with powdery mildew conidia, they were placed on the floor of an inoculation tower (80 × 25 × 25 cm, *h* × *l* × *w*). Plants with visible powdery mildew pustules on the leaves were then shaken out over the inoculation tower. The inoculated samples were removed from the inoculation tower after 1 h and incubated at 20 °C on moist paper in a Petri dish for 16 h in the dark. Afterward, the inoculated leaf pieces were placed on paper soaked in ethanol/acetic acid (3/1, v/v) to decolorize. After bleaching (≈24 h), the leaf pieces were then transferred to papers soaked in lactoglycerol (lactic acid/glycerol/H2O, 1/1/1.3, v/v/v) for ≈3 h to fix the structures. Subsequently, the fungal structures were stained with Trypan blue (Sigma‐Aldrich, St. Louis, USA, 0.05% in lactoglycerol) by direct dripping.

The germination success on the leaf pieces and the test systems was analyzed using a digital microscope (VHX‐600 DSO, Keyence, Osaka, Japan). Germ development was divided into the following developmental stages: nongerminated (ng), formation of a primary germ tube (1gt), formation of a secondary germ tube (2gt), abnormal development (three or more germ tubes, abn), formation of an appressorial germ tube (agt), and the development of an appressorium (app). On each sample, 100 individual conidia were counted (*n* = 10). Conidia in a chain or in accumulations were not counted. Burst conidia were evaluated as turgid conidia, provided that the developmental stage was clearly recognizable.

### 2.7 Statistical Analysis

All results are given as mean values ± the standard deviation (wax analysis: *n* = 6, wetting analysis of leaves and artificial surfaces: *n* = 15, analysis of conidia development: *n* = 10). The data were statistically analyzed (*p* < 0.05) with the software R Studio (R Core Team (2021). R: A language and environment for statistical computing. Vienna, Austria. URL https://www.R-project.org/). The graphs were created with R studio and Sigmaplot (version 13.0, Systat Software GmbH, Erkrath, Germany). The normal distribution and the homogeneity of the variances of data were assessed with graphical methods (boxplots and QQ‐plots) or with the Shapiro–Wilk test and the Levene test. For the statistical evaluation of the different varieties and of the germination development on the different surfaces, an analysis of variance (ANOVA) followed by a post hoc Tukey test was performed. If the requirements for a parametric test were not met, a Box–Cox transformation was performed for the wax analysis data. Other data in these cases were tested with the Kruskal–Wallis test followed by a Dunn–Bonferroni post hoc test. To detect significant differences between the samples and the positive and negative controls in the inoculation experiments, the Dunnett test was used.

## 3 Results

### 3.1 Morphology of Wax Structures

#### 3.1.1 Morphology of Natural Wax Structures

**Figure 1:**
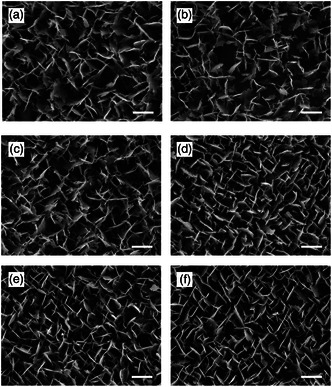
SEM images of the wax platelets of the adaxial and abaxial leaf sides (leaf 3) of the different wheat varieties of the greenhouse culture: a,c,e) adaxial leaf side; b,d,f) abaxial leaf side; a,b) Ponticus; c,d) Porthus; e,f) Akteur; scale bar: 1 μm.

Wax platelets were present on the adaxial and abaxial leaf sides of all three investigated wheat varieties. The platelets had irregular edges and were very dense (**Figure** **1**).

#### 3.1.2 Morphology of Recrystallized Structures

**Figure 2:**
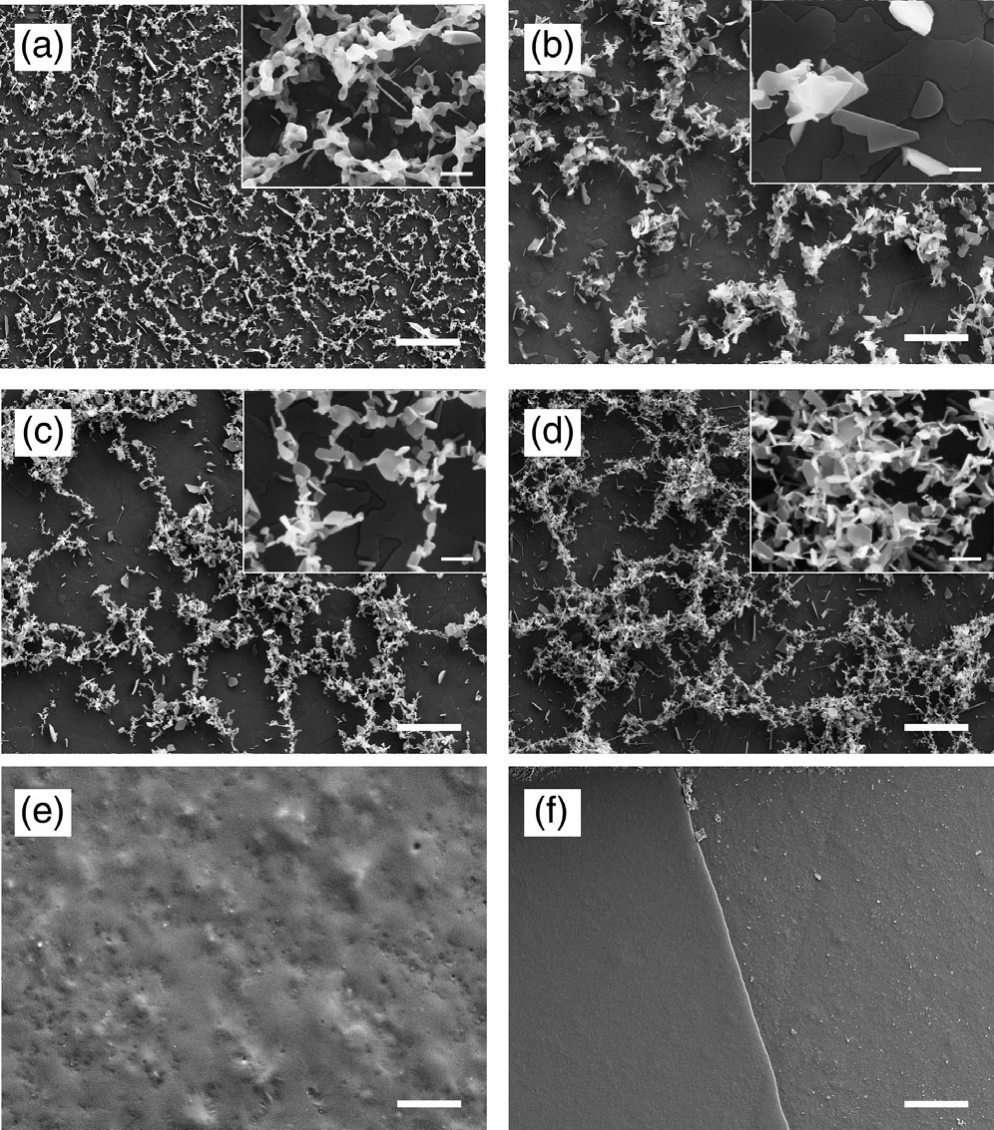
SEM images of recrystallized structures. a) Recrystallized wax, meshwork of granular structures with single scales in between; b) recrystallized 1‐octacosanol, accumulations of full‐edge platelets; c) recrystallized 1‐hexacosanal‐1‐octacosanol‐mixture, network of entire platelets, d) recrystallized 1‐octacosanal‐1‐octacosanol‐mixture, network of entire marginal platelets; e) wax layer on replica after melting of wheat wax; f) edge of wax coating to replica after removal with collodion, left wax layer, right pure replica. After vapor deposition, specimens were heated to 100 °C and subsequently cooled down to 4 °C. Samples were investigated three days after coating. Scale bars in (a–e) : 10 μm; scale bar detail: 1 μm; scale bar in (f): 100 μm.

The SEM examination of the test systems showed a uniform coating of the substrates. 3D, recrystallized structures were found on all test systems, except the melt test system. On the wax test system, granule‐like structures were formed, which were interconnected and formed a mesh (**Figure** **2**a). The recrystallization of 1‐octacosanol resulted in the formation of entire platelets and scales. The platelets accumulated partially; vertical scales were formed on flat‐lying platelets (Figure 2b). The recrystallized structures of the 1‐hexacosanal and 1‐octacosanal‐alcohol mixtures were the same. On the test systems that were vapor deposited with the aldehyde–alcohol mixtures, platelets with entire edges formed. They were very similar to the structures formed by the recrystallization of the pure alcohol. The full‐edged platelets accumulated and were partially linked to each other to form chains (Figure 2c,d). The interrupted recrystallization after wax melting by cooling the samples did not lead to any 3D structures. The wax formed a layer that was clearly visible after it was partially stripped off with collodion (Figure 2e,f).

### 3.2 Wettability

#### 3.2.1 Wettability of Wheat Leaves

The wettability of the leaves of the different varieties was investigated to obtain information on the water availability for the spore germination on the leaf surfaces and to develop artificial test systems with similar wetting properties.

**Figure 3:**
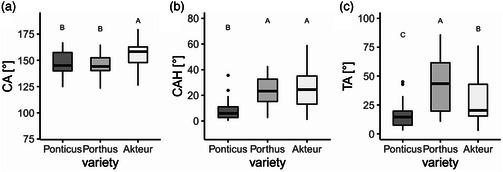
a–c) Wetting properties of the three wheat varieties: a) contact angle, b) contact angle hysteresis, c) tilting angle of water droplets (5 μL), measured on the leaf surface of leaf 3 of the respective wheat variety; the different letters indicate significant differences between the values measured on the different wheat varieties (*p* < 0.05, tested by a Kruskal–Wallis test followed by a post hoc Dunn test, *n* = 15).

The wheat leaves of the varieties were all hydrophobic or superhydrophobic, with CA around 150°. The CA of the variety Akteur (157.0 ± 11.9°) was higher than those of the other two varieties. The CAs of the varieties Ponticus (147.5 ± 11.7°) and Porthus (145.6 ± 10.3°) did not differ (**Figure** **3**a). The mean CAH of the wheat leaves was 19.2 ± 7.9°. The CAH was the lowest on the leaves of Ponticus (8.2 ± 8.3°). The CAH on the leaves of Porthus (23.1 ± 11.8°) and Akteur (26.2 ± 15.8°) were the same (Figure 3b). On average, the droplets on the wheat leaves rolled off at a tilt angle (TA) of 27.8 ± 10.4°. The TAs differed in every variety. The lowest TA was on the leaves of the variety Ponticus (15.7 ± 10.8°), the highest on the leaves of the variety Porthus (41.8 ± 23.3°). The TA of Akteur (28.2 ± 19.1°) was between the two other varieties (Figure 3c).

#### 3.2.2 Wettability of Artificial Surfaces

**Figure 4:**
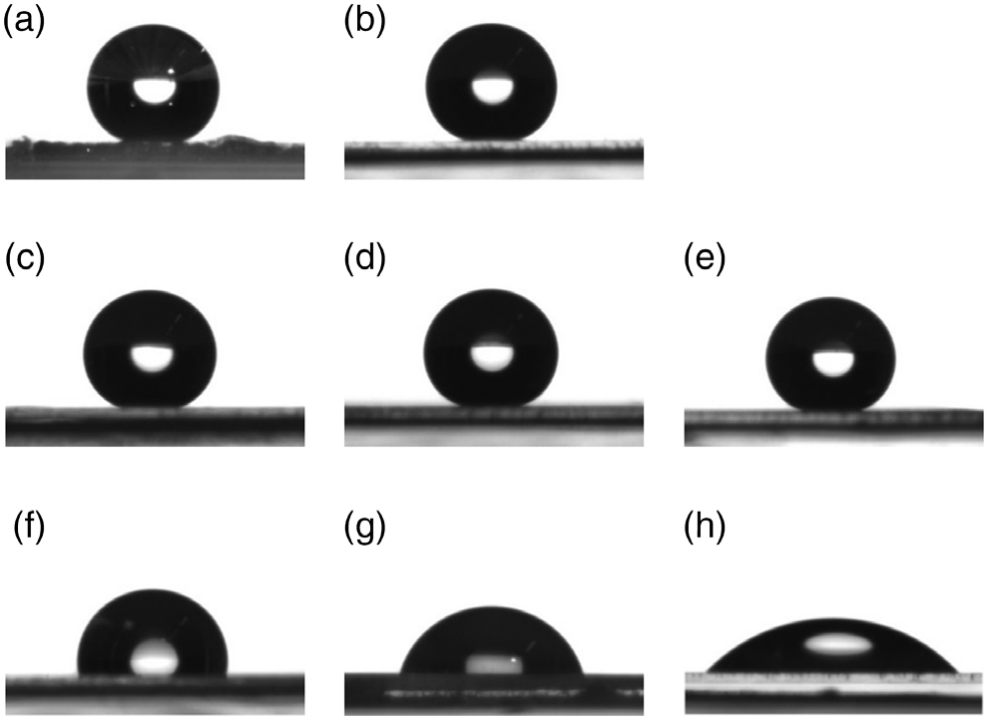
Goniometer photographs of water droplets on the different surfaces; a) wheat leaf, b) wax test system, c) C28OH test system, d) C26CHO test system, e) C28CHO test system, f) melt test system, g) replica, h) glass.

The wetting properties of the produced test systems were investigated in order to compare them with those of the wheat leaves. **Figure** **4** shows images of the water droplets on the test systems compared to water droplets on the wheat leaves and on uncoated glass and an uncoated replica used as negative controls.

**Figure 5:**
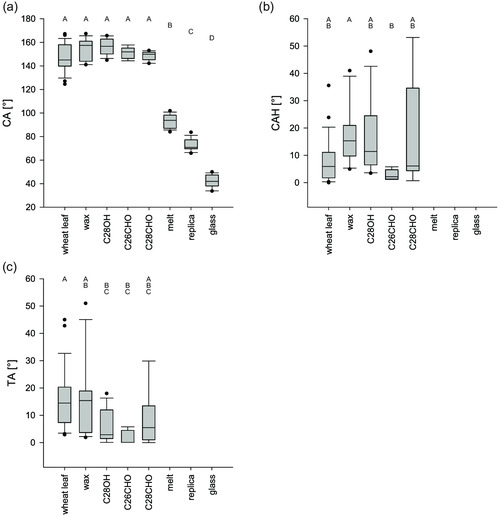
Wetting properties of the test systems. a) Contact angle (CA), b) contact angle hysteresis (CAH), c) tilting angle (TA) of the different surfaces (*n* = 15); the different letters indicate significant differences in CA, CAH and TA of the different surfaces (*p* < 0.05; (a) tested by ANOVA followed by post hoc Tukey test; (b,c) tested by Kruskal–Wallis test followed by post hoc Dunn Bonferroni test, *n* = 15).

The test systems with 3D structures had all CAs close to 150°. The CAs of the wax test system (154.7 ± 8.8°), the alcohol test system (156.3 ± 7.0°), and the two aldehyde test systems (C26CHO: 151.0 ± 5.0°, C28CHO: 148.7 ± 3.9°) did not differ from that of the wheat leaf (147,5 ± 11,7°). The CA of the C28OH test system was a bit higher than that of the wheat leaf. The melt test system without 3D structures had a lower CA than the other test systems and the wheat leaves. The CA of the melt test system was with 93.0 ± 5.6°, just in the lower hydrophobic range. The CA of the melt test system was also different from those of the two negative controls. Both the replica (72.9 ± 5.1°) and the glass (42.2 ± 5.5°) had hydrophilic surfaces (**Figure** **5**a).

On the melt test system, as on the negative controls, the droplets did not roll off. Therefore, neither CAH nor TAs could be measured. On all selected test systems with structure, the CAH was the same as on the wheat leaf. The CAH of the C26CHO test system was lower than that of the wax test system, at 2.9°± 1.9° (Figure 5b). The CAH of the C28CHO test system (19.4 ± 20.5°) was not different from that of the 1‐octacosanol test system (16.2 ± 13.1°) or the wax test system (28.0 ± 8.1).

On the wax test system, the same TA (14.9 ± 13.8°) was found as on the wheat leaf (15.7 ± 15.1°). The TA of the C28CHO test system (8.8 ± 9.9°) was also not statistically different from the wheat leaf. On the C28OH test system (5.7 ± 6.1°) and the C26CHO test system (1.5 ± 2.5°), the droplets rolled off at a lower TA than on the wheat leaf. The TAs of both aldehyde test systems did not differ from that of the C28OH test system (Figure 5c).

### 3.3 Wax Composition

**Figure 6:**
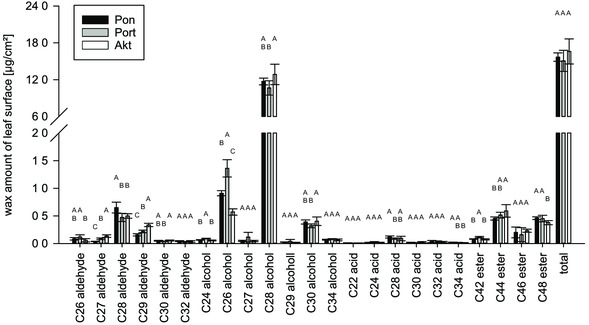
Wax compositions of the different wheat varieties of the field cultures: the different letters indicate significant differences between the amounts of the respective wax component (Pon: Ponticus, Port: Porthus, Akt: Akteur; *p* < 0.05, tested by ANOVA followed by post hoc Tukey test, *n* = 6).

The composition of the epicuticular waxes of the three different wheat varieties Ponticus (low susceptibility), Porthus (medium susceptibility), and Akteur (high susceptibility) was compared to investigate whether there were variety‐specific differences in wax chemistry. The total wax amounts of the three varieties Ponticus (15.68 ± 0.68 μg cm^−2^), Porthus (15.06 ± 1.72 μg cm^−2^), and Akteur (16.61 ± 2.05 μg cm^−^^2^) were the same. The wax of all three varieties consisted with 84% mainly of alcohols. The main component in each case was 1‐octacosanol (70.8–77.4%). In the wax of the Porthus variety, the C_28_ alcohol content was with 10.65 ± 1.17 μg cm^−^^2^ lower than in the wax of the Akteur variety with 12.86 ± 1.66 μg cm^−^^2^. The C_28_ alcohol content of the variety Ponticus was with 11.71 ± 0.54 μg cm^−^^2^ not different from any of the other two varieties. Esters and aldehydes constituted a minor part of the wheat wax. Their proportions were less than 10% each and did not differ between the varieties. The C_28_ aldehyde 1‐octacosanal constituted 3.0% (Akteur) to 4.2% (Ponticus) of the total wax. The absolute amounts of C_28_ aldehyde were clearly below 1 μg cm^−^^2^. In the wax of the variety Ponticus, the C_28_ aldehyde content was with 0.65 ± 0.10 μg cm^−^^2^ higher than in the wax of the variety Porthus with 0.47 ± 0.07 μg cm^−^^2^ and in the wax of the variety Akteur with 0.50 ± 0.04 μg cm^−^^2^. The proportions of the C_26_ aldehyde 1‐hexacosanal were below 1%. The maximum amounts were 0.12 ± 0.04 μg cm^−^^2^ in the wax of the Porthus variety. The lowest amount of C_26_ aldehyde was detected in the wax of the variety Akteur with 0.06 ± 0.03 μg cm^−^^2^. The amount of the C_26_ aldehyde in the wax of Ponticus (0.09 ± 0.02 μg cm^−^^2^) did not differ from the two other varieties. Acids were only detected in traces below 0.3 μg cm^−^^2^. They formed the smallest functional group of the wheat wax with 1.1–1.5% (**Figure** **6**).

### 3.4 Inoculation Experiments

#### 3.4.1 Inoculation Experiments on Wheat Leaves

**Figure 7:**
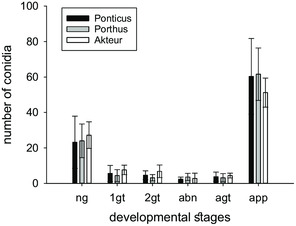
Germination development of *B. graminis* conidia on the leaf surfaces of different wheat varieties; ng: not germinated, 1gt: primary germ tube, 2gt: secondary germ tube, abn: abnormal development, agt: appressorial germ tube, app: appressorium; 100 conidia were counted in each case (*n* = 5).

The development of *B. graminis* f. sp. *tritici* was investigated on the three different susceptible wheat varieties. The germination behavior of the fungus showed no difference on leaves of the unsusceptible variety Ponticus and the more susceptible varieties Porthus and Akteur (**Figure** **7**). With ≈60 conidia, which corresponded to ≈80% of the germinated spores, most spores formed an appressorium. In all varieties, ≈1/4 of the conidia did not germinate. The number of remaining conidia was evenly distributed across the other developmental stages and was less than 10 in each of the varieties.

#### 3.4.2 Inoculation Experiments on Artificial Surfaces

**Figure 8:**
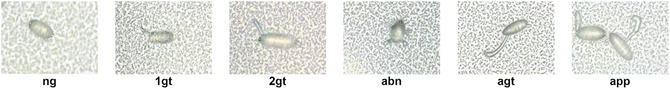
Developmental stages of *B. graminis* conidia on the wax test system; ng: not germinated, 1gt: primary germ tube, 2gt: secondary germ tube, abn: abnormal development, more than two germ tubes, agt: appressorial germ tube, app: appressorium; images 1000× magnified.

On all test systems, the different developmental stages of the powdery mildew conidia could be clearly identified. **Figure** **8** shows the different stages on the wax test system.

**Figure 9:**
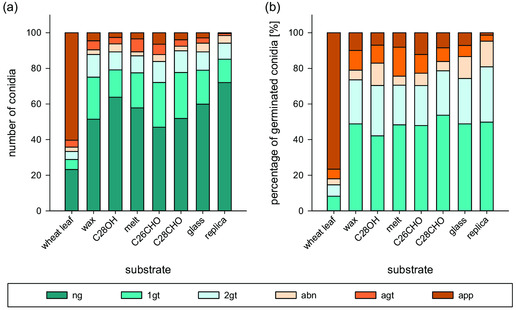
Germination development of *B. graminis* conidia on the wheat leaf and the test systems. a) Absolute number of conidia in the respective developmental stage; b) relative proportion of the individual developmental stages of germinated conidia; ng: not germinated, 1gt: primary germ tube, 2gt: secondary germ tube, abn: abnormal development, agt: appressorial germ tube, app: appressorium; the developmental stages of 100 conidia were determined in each case (*n* = 10).

The development of *B. graminis* was studied on the different test systems in order to identify possible germination‐inducing factors. The developments of the conidia on the test systems were similar (**Figure** **9**). On all test systems, the majority of the conidia did not germinate. The germination rates were maximum 53% on the C26CHO test system. The number of appressoria formed was not statistically different. On all test systems, only a few conidia (maximum 6 ± 3 on the C26CHO test system) formed an appressorium (Figure 9a). There was also no difference in the number of abnormally developed conidia and conidia with secondary and appressorial germ tubes. When looking at the development of germinated conidia, no significant differences were found between the test systems at all stages of development.

In comparison to the wheat leaves, a deviating development was observed on the test systems. With 77%, more conidia germinated on the wheat leaves than on all test systems. On the wheat leaves, the conidia that had formed an appressorium formed the largest proportion of the germinated conidia with 77 ± 13%. On the test systems, the biggest proportion (about half of the germinated conidia) remained in the first germ tube stage and more than 20% formed a secondary germ tube. Thus, the proportion of conidia with primary and secondary germ tube was higher than that on the wheat leaf with 8 ± 7% and 6 ± 3% (Figure 9b).

The development of conidia on the test systems and on the uncoated surfaces used here as negative controls were mostly the same. For instance, the number of appressoria formed on the wax (5 ± 3), C28OH (3 ± 3), C26CHO, and C28CHO test systems (7 ± 3 and 4 ± 3) differed from the number of conidia on the wheat leaf (60 ± 21), but not from that on glass (3 ± 3). Fewer appressoria also formed on the melt test system (4 ± 3) than on the wheat leaf and as few as on the replicate (0 ± 1). The number of germinated conidia on glass and the test systems did not differ either. Only on the melt test system germinated more conidia (42 ± 9) than on the replica (28 ± 12). There, more conidia also formed an appressorial germ tube (7 ± 5) than on the replica (1 ± 2). The number of appressorial germ tubes was otherwise the same on all surfaces.

## 4 Discussion

### 4.1 Morphology of Natural and Recrystallized Wax Structures

The cuticle and especially cuticular waxes confer specific properties to plant surfaces, which play a role in the development of phytopathogenic fungi. Since the leaf surface and the waxes deposited on it represent the first point of contact between a fungal spore and the plant, it was of particular interest which wax structures were present on the wheat leaves and whether the wax structures of susceptible and nonsusceptible varieties differed. Today, no study exists comparing the morphology of wax structures on wheat leaves with different susceptibilities to *B. graminis* f. sp. *tritici*. A study with barley mutants with less epicuticular wax structures showed a reduced germination capacity of *B. graminis* f. sp. *hordei* on the leaves of plants carrying the mutation compared to leaves of wild‐type plants.^[^ 85, 92 ^]^ No differences in wax morphology were found in the varieties examined here; all showed platelets and a comparable wax cover (Figure 1). In some studies, variety specific differences, which were shown by an altered wax morphology of the abaxial leaf sides, were found between blue‐green and non‐blue‐green or glossy varieties at later stages of development.^[^ 93, 94, 95, 96, 97, 98, 99, 100 ^]^ In contrast to the studies mentioned, the wheat plants examined here were still in the vegetative development phase at the time of the study and all leaves were green. The early developmental stage of the wheat plants was chosen here because, depending on the severity of the infestation, initial treatment with fungicides may be carried out at this stage.^[^ ^101^ ^]^

The test systems had a homogeneous coating and thus a uniform distribution of the substances and a uniform wettability, which was a crucial prerequisite for the following germination experiments. The recrystallized wax structures resembled the native wax crystals, but they did not correspond to them exactly. SEM images of the test systems showed that all of them had 3D structures with the exception of the melt test system, on which the formation of 3D structures was prevented by the melting process (Figure 2). The recrystallization into 3D structures proves again that wax crystals are formed by self‐assembly and that the main component 1‐octacosanol is considered to be the structure‐giving component, forming exclusively whole‐edged platelets.^[^ 44, 102, 103 ^]^

### 4.2 Wettability of Wheat Leaves and Artificial Surfaces

Relative humidity and surface moisture can have a significant influence on the establishment of fungal diseases in plants.^[^ ^104^ ^]^ Several studies with artificial substrates have shown that germination and differentiation of fungal spores can be stimulated by the hydrophobicity of the surface.^[^ 7, 17, 76, 105 ^]^ For example, *B. graminis* f. sp *hordei* only germinated on surfaces with CAs above 83° and with increasing CAs, the differentiation rate increased.^[^ ^76^ ^]^ The wheat leaves examined here had all hydrophobic, partly superhydrophobic surfaces. The CAs were between 145.6 ± 10.3° and 157.0 ± 11.9° (Figure 3) and are thus in agreement with previous studies.^[^ 53, 93, 106, 107 ^]^ The CAH and the TAs were low for all varieties, so that the droplets did not adhere strongly to the leaf surfaces and rolled off. The individual parameters of the wetting properties of the examined wheat varieties differed to some extent. However, the results did not indicate that their susceptibilities were due to differences in wetting behavior.

Artificial test systems were generated to mimic the wettability of the leaves. Despite the observed morphological differences between the 3D recrystallized wax structures on the test systems and the natural wax structures on the leaves, the wettabilities of the surfaces were similar. In contrast, the wettabilities of technical surfaces (e.g., silanized glass, FEP film) used in comparable studies were significantly higher than the wettabilities of the wheat leaves.^[^ 7, 9, 15, 76 ^]^ The melt test system also differs in its wettability from the plant surfaces, although it has the same chemical composition as the epicuticular waxes. Heating the samples above the melting temperature of the waxes resulted in a uniform wax layer, so that the wettability with a contact angle of only 93.0 ± 5.5° was clearly above that of the wheat leaves (Figure 5a). As on the negative controls, applied water droplets did not roll off. This proves how crucial the 3D wax structures are for the low wettability of the leaf surfaces.

### 4.3 Wax Composition of Wheat Leaves and Artificial Surfaces

The wax composition of the three different varieties was investigated to see if the wax compositions of susceptible and nonsusceptible varieties differed from each other. The results of this work showed no differences in wax chemistry that could be related to the different susceptibilities (Figure 6). The total wax amounts and the rankings of the substance classes did not differ for all three varieties. The wheat wax consisted of alcohols, aldehydes, acids, and esters. The C_28_ alcohol 1‐octacosanol was the main constituent of the wax. The results are thus consistent with previous studies on the wax composition of wheat.^[^ 44, 83, 93, 108 ^]^ Differences between the varieties were found in nonrelevant substance groups such as acids and esters. These substance classes were only detected in very low amounts below 0.5 μg cm^−^^2^, and no signaling or structure‐forming function is attributed to them.^[^ 44, 83 ^]^ Their minor differences did not indicate any correlation with the degree of susceptibility. A successful germination represents the beginning of a powdery mildew infection. Various studies have shown that aldehydes and especially the C_26_ aldehyde and the C_28_ aldehyde promote the germination of *B. graminis*.^[^ 75, 76, 78, 83, 84, 85, 88, 89, 90 ^]^ This suggests that the wax of susceptible varieties may contain more signaling components such as C_28_ aldehyde or C_26_ aldehyde than that of less susceptible varieties, resulting in increased germination that ultimately leads to more severe disease course. This hypothesis could not be confirmed here. The highest amount of C_28_ aldehyde was found in the least susceptible variety Ponticus. The amounts of the C_26_ aldehyde were the same in the most susceptible variety Akteur and in the least susceptible variety Ponticus (Figure 6). Furthermore, the wax of the most susceptible variety had the lowest proportion of the C_26_ alcohol for which an inducing effect is also described.^[^ ^83^ ^]^ These results suggest that susceptibility is not manifested in differences in these single wax components. Similar germination tests of *B. graminis f. sp. tritici* on wheat and on the non‐host‐plant barley showed no difference in germination success despite slightly different wax compositions.^[^ ^83^ ^]^ From this it can be deduced that only larger differences in wax chemistry influence germination behavior, or that the wax composition is not decisive for germination success. Carver and Thomas^[^ ^82^ ^]^ also assumed, after experiments with different f. sp., that nonspecific properties of the cuticle are decisive for recognition.

By coating an artificial surface with wheat wax, a test system was produced that exhibited the wetting properties and chemical information of the epicuticular waxes of wheat leaves. Its chemical stability during the evaporation process was demonstrated in a previous study.^[^ ^51^ ^]^ It was also shown that the wax coating of the artificial surface is not only qualitatively but also quantitatively equivalent to the natural wax coating.

### 4.4 Inoculation Experiments on Natural and Artificial Surfaces

It was tested whether the different susceptibilities of the different varieties could be recognized in the germination behavior. No differences were found: the same number of conidia germinated and differentiated on the leaves of all three varieties (Figure 7). This is in agreement with previous studies investigating germination behavior with compatible and noncompatible host–pathogen systems.^[^ 70, 109, 110 ^]^ In the early phase of pathogenesis, before penetration, no differences were found there either, but more haustoria were formed on compatible host–pathogen systems than on incompatible ones.^[^ ^109^ ^]^ Our results do not indicate that the resistance of the wheat varieties is due to a reduced germination capacity or a lower differentiation of the fungus. Rather, various post‐infection defence mechanisms must obviously contribute to the development of resistance.^[^ ^19^ ^]^

Because the natural leaf surfaces did not show any variety‐specific differences in wax morphology, wax chemistry, or wettability of the leaves, it was not necessary to differentiate between varieties in the in vitro tests. Our results show that germination behavior on the different test systems was similar. However, it was clearly different from that on the wheat leaf. On the host plant leaves, germination rates were high and most conidia formed an appressorium. On the test systems, most conidia had not germinated. The conidia that germinated developed a primary and secondary germ tube but hardly any appressorial germ tubes or appressoria (Figure 9). The detailed discussion of the individual test systems follows in the next sections.

#### 4.4.1 Influence of Wettability on Germ Development

On substrates that had a higher wettability than the wheat leaves (glass, melt test system), the development of the conidia was not inhibited compared to substrates that had a similar wettability as the wheat leaf (C28OH test system, wax test system). Contrary to expectations, the hydrophobicity did therefore not seem to have a positive effect on the germination behavior. Earlier studies had indeed shown differences in germination in relation to the substrate wettability. On hydrophilic surfaces such as agarose or glass, fewer conidia germinated, and there was no formation of appressoria.^[^ 9, 15, 70, 76 ^]^ These surfaces are usually referred to as noninductive surfaces.^[^ ^80^ ^]^ On less wettable surfaces, such as silanized glass or FEP films, increased germination and appressoria formation could be observed.^[^ 9, 15, 76 ^]^ In previous studies, the substrates used as hydrophobic surfaces were significantly less hydrophobic than the natural surface.^[^ 7, 9, 15, 76 ^]^ If the hydrophobicity of the leaf surface is a decisive factor for germination development, considerably more conidia at an advanced stage of development would have been expected on the C28OH test system, which is as un‐wettable as the leaf. Our data show that the hydrophobicity of the plant surface alone has no inductive effect on the germination development of *B. graminis*. We also exclude an inhibitory effect of the C_28_ alcohol, as it is the main component of the wheat wax. Moreover, an earlier study with the C_28_ alcohol had shown no effect on the germination behavior of *B. graminis*.^[^ ^83^ ^]^

#### 4.4.2 Influence of the Wax Chemistry on the Germ Development

The development of the conidia on the melt test system, which exclusively mimics wax chemistry, did not indicate that the wax chemistry is a decisive signal factor. Although the germination rates on the melt test system were about 15% higher than on the pure replica, more than half of the conidia did not germinate on the melt test system either. The number of conidia on the melt test system and on the replica that formed an appressorium did not differ from each other. The effect of wax chemistry on germination development is evaluated differently in the literature. Like the results of the present studies, the results of some earlier studies argue against an influence of wax chemistry. For example, the development of conidia on leaves and epidermis strips was not affected after removal of the epicuticular waxes.^[^ 7, 15, 76, 82, 111, 112 ^]^ Other studies describe an influence of the specific wax composition on the germination development of *B. graminis*. Some authors suspected an inhibiting effect of specific wax substances and structures,^[^ ^113^ ^]^ some suggested an stimulating effect: Studies with wax mutants and different natural surfaces with and without waxes indicated a crucial role of wax chemistry in host recognition. On the leaves of the wild‐type plants and the natural surfaces with waxes, appressoria formation was higher than on the leaves of the plants with altered wax chemistry and on the natural surfaces without waxes.^[^ 70, 86 ^]^ However, not only the wax chemistry but presumably also the wetting of the leaves was altered. In earlier studies, in contrast to the present study, an induction of germination and differentiation by wax chemistry alone could also be detected on artificial substrates.^[^ 70, 75, 77, 88 ^]^ The development of the conidia was more progressed on the waxy surfaces than on glass. Nevertheless, it remained behind the development of the conidia on the leaves of the host plant. Other factors of the leaves, which were not represented by the wax extracts, therefore seem to be responsible for the complete development of the fungus.

#### 4.4.3 Influence of Long‐Chain Aldehydes on Germ Development

In particular, the positive effect of long‐chain aldehydes on the germination behavior could be shown.^[^ 75, 78, 83, 84, 85, 89, 90 ^]^ Zabka, et al.^[^ ^76^ ^]^ found in in vitro experiments on different surfaces coated with the C_26_ aldehyde that the development of the fungus was more stimulated on the surfaces with a lower wettability than on the surfaces with a higher wettability. The authors suspected that surfaces with lower wettability would stimulate differentiation even more. In later experiments with a further developed in vitro test system, the effect of various very long‐chain aldehydes was investigated in conjunction with a lower wettability. The differentiation rates on the test system spiked with C_26_ aldehyde were higher than those in the study by Zabka, et al.^[^ ^76^ ^]^ However, most of the differentiated conidia remained in the appressorial germ tube stage. The wettabilities achieved with the sophisticated test systems used in previous studies were still below the wettability of the natural leaf surface. With the C26CHO and C28CHO test systems developed in the present study, the signaling effect of the C_26_ and C_28_ aldehydes on the prepenetration process could be investigated for the first time in combination with a wettability as low as on the leaf. The results of the germination experiments could not confirm the stimulating effect of the aldehydes. The lower wettability in comparison to the other studies showed no positive effect on germination development. The germination rates were the same (C28CHO test system) or even lower than on glass (C26CHO test system). Appressoria formation was not increased by the aldehydes and the low wettability. As on glass, only a few conidia developed an appressorium on the aldehyde test systems (Figure 9). In contrast to many previous studies, the signal effect on *B. graminis* f. sp. *trititci* (Bgt) and not on *B. graminis* f. sp. *hordei* (Bgh), the powdery mildew of barley, was investigated here. The main aldehyde of wheat wax is the C_28_ aldehyde, in barley it is the C_26_ aldehyde (Figure 6).^[^ 44, 75, 83 ^]^ Nevertheless, both *formae speciales* (f. sp.) also germinate and differentiate on the respective non‐host‐plant and the different f. sp. do not seem to be adapted to a specific aldehyde spectrum.^[^ 70, 83, 88 ^]^ There is no clear evidence for coevolution between the different f. sp. and their host plants and it was suspected that specific wax components, such as the C_26_ aldehyde, are a general requirement for normal germination of powdery mildew conidia.^[^ 85, 114, 115 ^]^ In both Bgh and Bgt, the positive effect of C_26_ aldehyde on the germination of the fungus could also be shown in vivo. Spraying with aldehyde‐containing solutions caused the conidia to germinate to a similar extent as on the leaves of the wild‐type plants.^[^ 84, 89, 90 ^]^ In Bgh, a positive effect on the differentiation rates of aldehyde could thus also be shown.^[^ ^84^ ^]^ In the present work, however, no positive influence of aldehydes on the germination behavior of Bgt could be observed (Figure 9). Assuming that aldehydes have an inducing effect, this would mean either that the germination development in the present study was inhibited by another factor or that the substance was not accessible to the conidia. The wettability of the test systems used here was considerably lower than that of the test systems used in the past and that of the leaves of various wax mutants sprayed with aldehydes. However, it cannot be ruled out that other potentially stimulating substances, such as cutin monomers or cell wall components, were also released by spraying the plant surfaces with organic solvents. An inhibitory effect of the low wettability is highly unlikely, as it corresponds to the wettability of the natural leaf surface.

#### 4.4.4 Influence of Wettability and Wax Chemistry on Germ Development

Even in the in vitro studies that had shown a stimulatory effect of different surfaces, the appressoria formation rates remained behind those on the plant surface.^[^ 75, 76, 77, 78, 83 ^]^ This supports the thesis that fungi need a combination of signals in order to fully develop.^[^ ^116^ ^]^ In the past, no test system existed that fully represented both the chemical and the wetting properties of the leaf surface. The wax test system allowed to investigate the influence of wax chemistry and hydrophobicity in combination on the germination of *B. graminis* in vitro. The amount of wax applied also corresponded to that of the natural leaf surface.^[^ ^51^ ^]^ Nevertheless, germination and appressoria formation were not stimulated on this test system either (Figure 9). Apparently, the test system did not provide the necessary signals for germination development. Assuming that hydrophobicity and wax chemistry are decisive factors for the germination development of *B. graminis*, a significantly higher number of germinated and differentiated conidia would have been expected on the wax test system used here. However, the results of this work indicated that hydrophobicity and wax chemistry in combination are not decisive factors for the progression of *B. graminis* development. It should be noted here that the number of abnormally developed conidia was similar on all surfaces including the natural leaf surface. Although the primary germ tube had apparently not perceived an inductive surface on the artificial surfaces, nevertheless no more short subsidiary germ tubes had formed than on the wheat leaves, which presented an inductive surface.

## 5 Conclusions

No variety‐specific differences in wax morphology and wax chemistry were found in the investigated varieties. A relation between the individual wetting parameters of the three varieties and their susceptibility could not be identified. Accordingly, the germination experiments on the three wheat varieties did not show any differences in the prepenetration process of *B. graminis*. In order to identify possible germ‐inducing signals, in vitro test systems were successfully developed. The thermal evaporation of the substances produced a uniform coating of the technical surfaces, with or without 3D structures, depending on the recrystallization condition. The surface properties of the wheat leaves could thus be successfully imitated on a technical surface. In addition to the test system representing both the chemical properties of the waxes and the wettability of the plant surface, it was possible to realize test systems representing only selected properties of the leaf surface so that their influence on the prepenetration process of *B. graminis* could also be studied separately in vitro. The germination experiments on the test systems did not identify any decisive signals for the development of *B. graminis*. On the artificial surfaces, the prepenetration process was neither stimulated by the hydrophobicity nor by the wax chemistry. The combination of wax chemistry or aldehydes with low wettability also did not stimulate germination or differentiation. The question of which properties of the plant surfaces are the decisive signals for *B. graminis* therefore remains an open area of research. The here used thermal evaporation method provides a variable tool for investigating the influence of different factors on germination success. For example, the topography of the leaf surface can be included in the investigation by moulding the epidermis surfaces and subsequent vapor deposition, or isolated cuticles can be used as a substrate. Furthermore, the germination success of other phytopathogenic fungi can be investigated with the help of the test systems.

## Conflict of Interest

The authors declare no conflict of interest.

## Data Availability

The data that support the findings of this study are available from the corresponding author upon reasonable request.
